# CRISPR-based functional genomics tools in vertebrate models

**DOI:** 10.1038/s12276-025-01514-0

**Published:** 2025-07-31

**Authors:** Gaurav K. Varshney, Shawn M. Burgess

**Affiliations:** 1 Genes & Human Disease Research Program, Oklahoma Medical Research Foundation, Oklahoma City, OK USA; 2 Translational and Functional Genomics Branch, National Human Genome Research Institute, National Institutes of Health, Bethesda, MD USA

**Keywords:** Genetic engineering, Mutagenesis

## Abstract

The advent of CRISPR–Cas technologies has revolutionized functional genomics by enabling precise genetic manipulations in various model organisms. In popular vertebrate models, including mice and zebrafish, CRISPR has been adapted to high-throughput mutagenesis workflows, knock-in alleles and large-scale screens, bringing us closer to understanding gene functions in development, physiology and pathology. The development of innovative technologies, such as base editors, capable of single-nucleotide modifications, and prime editors, offering precision edits without double-strand breaks, exemplifies the expanding toolkit. In addition to gene editing, transcriptional modulation, that is, CRISPR interference and CRISPR activation systems, can elucidate the mechanisms of gene regulation. Newer methods, such as MIC-Drop and Perturb-seq, which increase screening throughput in vivo, hold significant promise to improve our ability to dissect complex biological processes and mechanisms. Furthermore, CRISPR-based gene therapies for treating sickle cell disease and other monogenic diseases have already demonstrated their potential for clinical translation. Here this Review covers the transformative impact of CRISPR-based tools in vertebrate models, highlighting their utility in functional genomics research and disease modeling.

## Innovative CRISPR tools revolutionize the study of gene function

Recent advances in DNA sequencing have allowed scientists to gather vast amounts of genetic data, but understanding what those sequences actually do requires additional tools that allow scientists to manipulate those sequences. This Review explores the impact CRISPR–Cas technology has had on the field of functional genomics, which aims to understand gene functions and their impact on health and disease. Functional genomics uses various biological data types, such as DNA sequences, chromatin structure, RNA expression and measures of protein function, to study how small changes in genomic sequence create observable differences between animals of the same species, including human beings. CRISPR–Cas technology is a tool that allows precise editing of sequences in the genome, including the sequences containing genes and the sequences in between those genes that control gene expression. CRISPR–Cas has revolutionized the study of gene functions by enabling targeted mutations in organisms such as zebrafish and mice. The study discusses how CRISPR–Cas can create gene ‘knockouts’ and ‘knock-ins’. It also explores new technologies such as base and prime editing, which allow changes to single DNA bases with high precision. These tools are rapidly advancing our understanding of genetics and have potential therapeutic applications that are already being tested in the clinic.

This summary was initially drafted using artificial intelligence, then revised and fact-checked by the author.

## Introduction

Rapid advances in sequencing technologies have greatly improved our ability to generate massive amounts of genomic data. This avalanche of data is only the first step in the true goal of biologists, to completely predict phenotype based on the genotype. To address this challenge, we need exponentially more functional data. Researchers responded by creating the field of functional genomics, which leverages data from multiple biological modalities (genome sequences, transcriptomes, epigenomes, proteomes, metabolomes and so on) to better understand how genetic variation can change an organism at the level of protein functions, gene regulation or other complex genetic interactions. We still do not know the function of every gene in an organism’s genome, nor can we robustly predict the impact of genetic variation in either coding regions or in the regulatory regions of genes.

For example, the human genome is estimated to have approximately 20,000 protein-coding genes and perhaps an equal number of noncoding genes^1^. Approximately 70% of these genes have been assigned a function either by direct enzymatic assays, biochemical characterization, prediction based on homology or direct genetic demonstration of function. That leaves approximately 6000 genes that are currently completely uncharacterized. There are two further complications. First, clinical sequencing of patients with genetic diseases finds variants in human genes that are impossible to predict whether they are pathogenic (variants of uncertain significance) at a rate 2.5 times higher than ones they can interpret^2^. One study showed that over a 10-year period, only 7.7% of these variants of uncertain significance were reclassified after further data^3^. This bottleneck needs to be addressed with new approaches.

Second, over the past 15 years, an enormous effort has been placed into genome wide association studies^4^. This is a powerful approach that can map genetic influences for any measurable human trait given a large enough sample size. However, early in the efforts, it became clear that ≈95% of the identified ‘risk’ small-nucleotide variants were in noncoding regions of the genome^5^. Most of these genomic regions, including tens of thousands of variants, have not been functionally tested.

This is the fundamental challenge of functional genomics: to provide us with the systematic perturbation of genes and/or regulatory regions and analyze the ensuing phenotypic changes at a scale that can inform us about both basic biology and human pathology. Most of the published efforts are carried out in cell culture, but many questions about development, physiology or tissue homeostasis cannot be addressed in monolayer cultures (or even organoids), so various model organisms must also be utilized.

In the past, the most common approach was to test individual genes in a biological process by inactivation (that is, mutation), either randomly through ‘forward’ genetics or targeted through ‘reverse’ genetics. However, the massive amounts of genetic and genomic multimodal data currently being generated both allow for and even require high-throughput screening methodologies to make sense of all the data. Researchers are developing massively parallel functional interrogation of genes and proteins by employing large-scale assays. Essential for these approaches to work is a simple and efficient method for targeting and mutating the genome in cell culture or in vivo. The discovery of CRISPR–Cas opened a new era in functional genomics and has given us a powerful and easily reprogrammable tool for targeted genome editing.

**Fig. 1:**
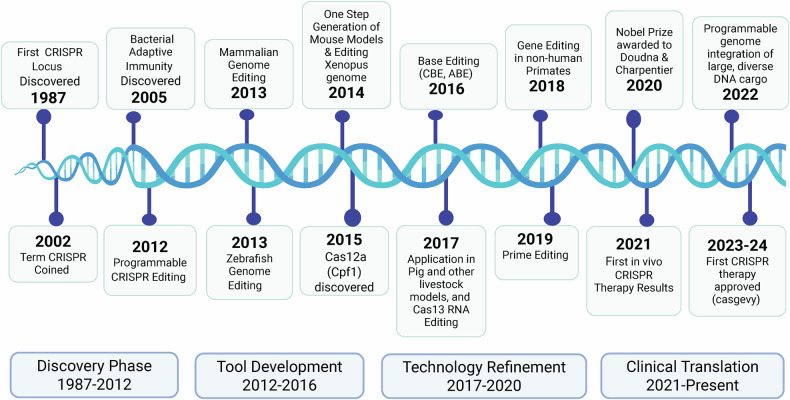
Timeline of key milestones in CRISPR genome editing research and clinical development. The progression of CRISPR research is divided into four major phases. This timeline illustrates the rapid evolution from basic discovery to clinical application, highlighting transformative innovations in genome and epigenome editing technologies. (Figure created using Biorender.com).

**Fig. 2:**
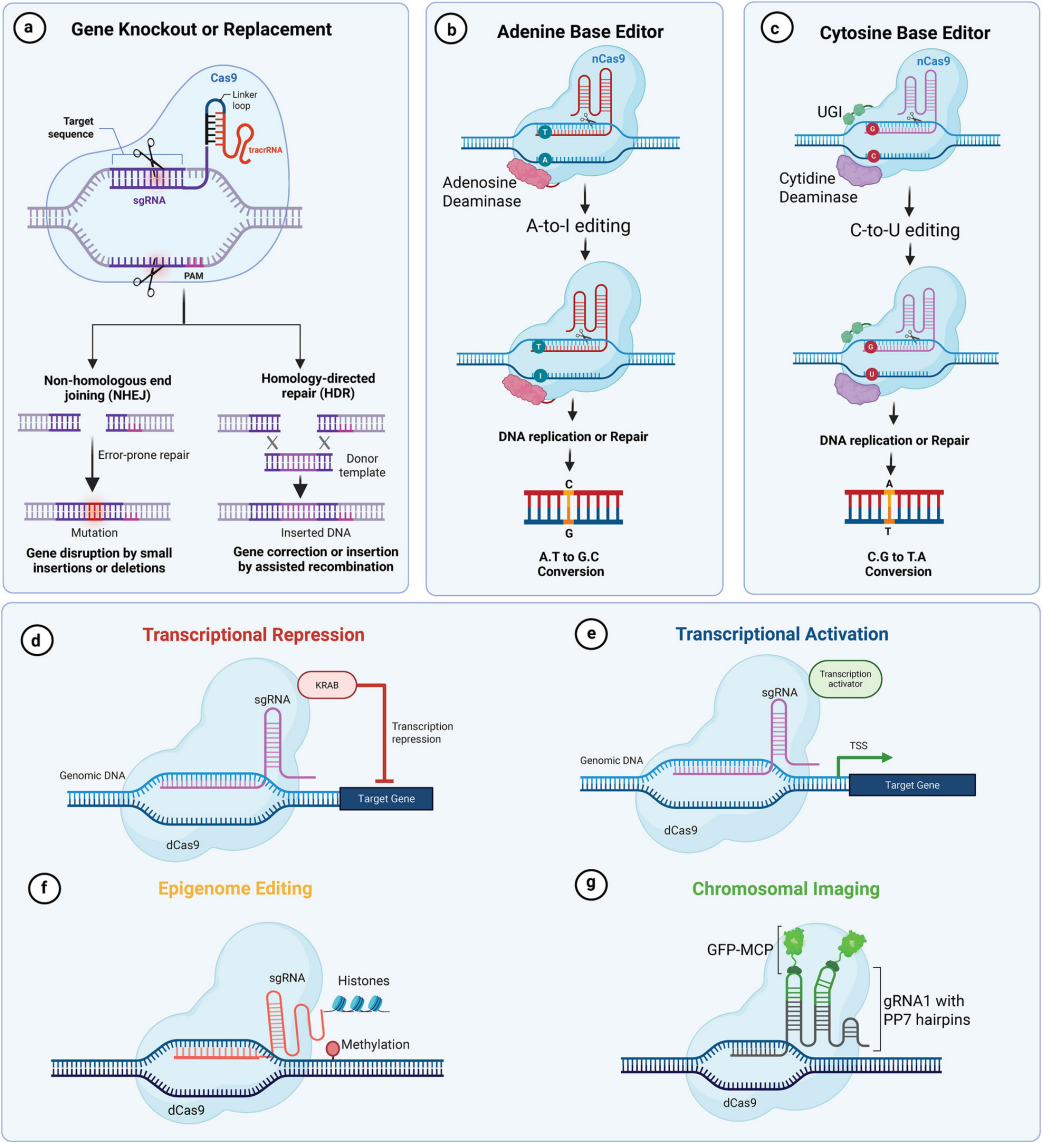
Overview of CRISPR-based genome and epigenome engineering tools. **a** Gene knockout or replacement: Cas9 nuclease creates DSBs at target sites guided by sgRNA. Repair occurs via NHEJ, leading to small insertions or deletions that disrupt gene function, or via HDR using a donor DNA template to enable precise gene correction or insertion. **b** ABE: a catalytically impaired Cas9 (nCas9) fused to an adenosine deaminase converts A•T base pairs to G•C through A-to-I (inosine) editing, which is read as G during DNA replication or repair. **c** CBE: nCas9 is fused to a cytidine deaminase and often paired with UGI. This enables C-to-T editing, converting C•G base pairs to T•A. **d** Transcriptional repression (CRISPRi): a catalytically dead Cas9 (dCas9) is fused to a KRAB repressor domain and guided to promoter regions, where it inhibits gene transcription without cutting DNA. **e** Transcriptional activation (CRISPRa): dCas9 is fused to a transcriptional activator and directed to a gene’s promoter or enhancer to initiate transcription. **f** Epigenome editing: dCas9 is tethered to epigenetic modifiers (for example, methyltransferases) to install or erase epigenetic marks such as DNA methylation at specific loci, without altering the underlying DNA sequence. **g** Chromosomal imaging: dCas9 is used in conjunction with guide RNAs bearing PP7 hairpins and fluorescent proteins (for example, GFP-MCP) to visualize specific genomic loci in live cells.(Figure created using Biorender.com).

CRISPR-based tools have enabled researchers to achieve once-unimaginable goals because of their simplicity, versatility and universality, and unlike customized DNA-binding protein systems, they do not require specialized expertise. CRISPR–Cas systems have been harnessed by geneticists in many ways and the field has grown significantly over the past decade (Fig. 1). By far its most common use is to precisely target specific genetic loci with double-stranded breaks (DSBs), facilitating the generation of knockout and knock-in alleles for studying gene function and disease modeling. Beyond gene editing, researchers have developed CRISPR–Cas9 applications for transcriptional modulation, epigenome editing, live imaging of the genome and lineage tracing and many other applications (Fig. 2). Ongoing efforts focus on and expanding the target ranges of Cas proteins and improving the specificity to minimize off-target effects. The development of ‘base editors’ and ‘prime editors’ allows for precise, single-nucleotide modifications. Base editing enables single-base substitutions without needing DSBs, while prime editing allows for targeted insertions and deletions. These innovative technologies can potentially improve the precision and efficiency of genome editing, expanding the possibilities for functional genomics research and therapeutic applications. CRISPR–Cas technology is driving innovation in genetics and research into therapeutics. This review provides a survey of recent advances in CRISPR–Cas genome editing technologies with an emphasis on their applications to functional genomics and in vivo editing of vertebrate model organisms.

## Targeted mutagenesis approaches

The rapid adoption of next-generation sequencing technologies in disease diagnostics has led to a surge in identifying novel candidate disease genes and variants. Most of these genes lack functional data and functional analysis is essential to understand their role in disease pathology. One approach to establishing disease causality is to target the candidate gene in a model organism and to demonstrate a phenotype similar to the one observed in the human gene. Historically, researchers relied on random mutagenesis (chemical, radiation or retroviral) or transient knockdown approaches such as RNAi or morpholinos to inactivate genes in model organisms, except for mice, where targeted gene inactivation was possible but required many months of work and highly specialized training. Later, customizable nucleases such as ‘zinc-finger nucleases’ (ZFN)^6^ or transcription activator-like effector nucleases (TALEN)^7^ were utilized to study gene function via targeted mutagenesis. These nucleases were very effective, but each new DNA sequence target required the proteins to be re-engineered, reducing the throughput of the technology.

Programmable nucleases typically create a DSB at the target site, which is usually repaired by the endogenous DSB repair mechanisms of the cell. The nonhomologous end joining (NHEJ), homology-directed repair (HDR), and microhomology-mediated end joining (MMEJ) pathways are all available mechanisms for the necessary DNA repair. NHEJ is the default pathway in vertebrates and often leads to small insertions or deletions at the DSB site, resulting in gene knockouts. HDR induces targeted DNA changes by supplying a repair template, which is recombined into the damaged locus. MMEJ is often utilized to create larger deletions or for precise knock-ins by leveraging the tendency of small repeat regions to align during repair. The NHEJ pathway is the most used repair pathway by researchers. It is technically simple (causes a DSB and lets the cells do the rest) and typically results in small nucleotide insertions or deletions that cause frameshifts, making it suitable for rapidly generating knockouts. Meanwhile, MMEJ can be used for creating larger deletions but is more commonly applied for precise knock-ins, with the researchers providing a template containing short homology sequences to the sequences flanking the cut site. This reaction is much less efficient but provides opportunities for integrating DNA sequences with various ‘useful’ functions^8^.

The CRISPR–Cas system was initially discovered in bacteria and archaea and is a family of endonucleases used to protect organisms from viral infection^9^. Once the biochemical activity (that is, sequence-specific, double-stranded nuclease) was determined for the Cas proteins, they were quickly repurposed for genome editing, and they are remarkably efficient across diverse organisms. Among all the CRISPR families identified, the CRISPR–Cas9 system originating from the bacterium *Streptococcus pyogenes* has been one of the most commonly used genome editing tools. The CRISPR–Cas9 system utilizes a guide RNA (gRNA) with a stretch of 20 nucleotides that target a specific DNA sequence through complementary base pairing, and once that DNA target is found, the protein catalyzes a DSB^9^.

Following the seminal publication by Jinek et al. in 2012 showing that the Cas9 protein from *S.* *pyogenes* could be directed by guide RNA to cleave DNA at specific sites^10^, establishing a mechanism for targeted genome editing, three other studies demonstrated the use of CRISPR–Cas9 was extremely effective at targeting loci in the genome of human and mouse cells^11–13^.

Since the original publications, generating knockouts in the genes of interest in different organisms has become routine in laboratories and revolutionized functional studies. Hwang et al. first demonstrated using CRISPR in zebrafish, achieving precise gene disruptions at the *tyr* and *gata5* loci^14^. They demonstrated that co-injection of Cas9 mRNA and single guide RNA (sgRNA) efficiently generated mutations, establishing a powerful tool for zebrafish genetics. Subsequently, Jao et al. demonstrated the biallelic disruption of multiple loci and efficient germline transmission, paving the way for the broader adoption of this technology in zebrafish^15^. Following initial publications, Gagnon et al. and Varshney et al. published methods to show the in vitro synthesis of sgRNAs, bringing down the cost and timeline for CRISPR mutagenesis^16,17^. We published the first large germline dataset in vertebrates, targeting 162 loci in 83 genes in the zebrafish genome, showing a 99% success rate for generating mutations and an average germline transmission rate of 28% (ref. ^17^). Since then, knockouts have been generated to study development, physiology and various human diseases. Owing to the scalability of this technology, many laboratories have utilized it to screen from tens to hundreds of genes in zebrafish; Pei et al. screened 254 genes to identify genes essential in the regeneration of hair cells and other tissues^18^ and Unal Eroglu et al. screened over 300 genes for their role in retinal regeneration or degeneration^19^. Similar approaches were used to search for genes associated with human diseases. For example, Thyme et al. targeted zebrafish orthologs of 132 human schizophrenia-associated genes^20^, Griffin et al. generated mutants for 40 genes associated with childhood epilepsies^21^ and other researchers have targeted genes involved in disease-associated complexes or pathways such as the DNA repair pathway^22^, Fanconi anemia pathway^22^ and the survival of motor neurons (SMN) complex^23^.

The first use of CRISPR in mice was demonstrated by Shen et al. targeting an endogenous eGFP locus by co-injecting gRNA with ‘humanized’ Cas9 mRNA into one-cell embryos, achieving a gene disruption efficiency of 14–20%^24^. Subsequently, Wang et al. highlighted the remarkable ability of CRISPR–Cas9 to target single or multiple genes, and over the past decade, numerous knockouts have been generated in mice and rats. Large consortia, such as the Knockout Mouse Project, also adopted CRISPR technology and generated over 1,200 mouse models using the approach^25^. Apart from single-gene knockouts, CRISPR is also being adopted for large-scale genome-wide in vivo screens in mice. The majority of large-scale CRISPR–Cas gene knockout screens are performed in cell culture (or organoids), but in vivo screens can provide a more physiologically relevant context for studying gene function and such screens have yielded valuable insights into a wide range of biological processes and diseases.

A genome-wide CRISPR screen in a mouse model of non-small-cell lung cancer identified several genes whose loss-of-function mutations drive tumor growth and metastasis^26^. Another screen used a single mouse liver to uncover the regulation of hepatocyte function by using a library of sgRNAs targeting over 13,000 protein-coding genes to the liver to efficiently knock out genes in hepatocytes and identify genes that are essential for liver function^27^. A rat model of multiple sclerosis was used to identify genes that regulate T cell migration to the central nervous system^28^. Recently, researchers developed an innovative inducible mosaic animal for perturbation (iMAP) system. iMAP enables researchers to study the functions of multiple genes simultaneously across various tissues. It utilizes a germline-transmitted transgene carrying an extensive array of individually floxed, tandemly linked gRNAs that allows investigation of parallel analysis of gene functions in vivo^29^.

## Biallelic genetic disruption for rapid functional analysis

Generating stable homozygous mutant lines using CRISPR–Cas9 required breeding first generation (F0) mosaic animals to obtain stable lines in subsequent generations, which can take months or years, depending on the organism. Another approach is to breed two mosaic founders and screen for phenotypes in first filial generation (F1) generation in compound heterozygous animals, but this approach still requires animals be raised to sexual maturity and screening takes place in the subsequent generation. These time-consuming approaches do not scale sufficiently to address the growing number of novel candidate disease genes being identified. However, researchers have optimized the conditions for very efficient CRISPR F0 mutagenesis in several species and this offers a faster and more efficient alternative by enabling the direct analysis of phenotypes in the injected embryos or F0 generation animals, reducing the timeline from months or years to just days or weeks. This approach is particularly valuable for studying early developmental processes, analyzing complex phenotypes, knocking out multiple genes simultaneously and conducting large-scale genetic screens.

The high efficiency of CRISPR–Cas9 in inducing biallelic mutations in the F0 generation of mice, zebrafish and *Xenopus* makes it a promising tool for studying gene function in these model organisms. CRISPR F0 mutagenesis in zebrafish typically involves injecting a mixture of Cas9 protein or mRNA and sgRNA into one-cell stage embryos. The first example of generating biallelic mutation in zebrafish was using Cas9 mRNA and sgRNA, but the efficiencies varied significantly from locus to locus^15^. Since then, several strategies have been developed to enhance the efficiency and consistency of CRISPR F0 mutagenesis in zebrafish. Burger et al. demonstrated that injecting Cas9 protein and sgRNA as riboprotein complexes together with 1 M KCl to improve the solubility of the complex can achieve high targeting efficiencies, and phenotypes can be robustly observed in the F0 generation (named ‘Crispants’) with high penetrance^30^. Another technical improvement involves using multiple sgRNAs targeting different regions of the same gene. This increases the probability of generating biallelic mutations and reduces mosaicism^31^. Another strategy utilizes chemically synthesized CRISPR–Cas9 dual-guide ribonucleic protein (dgRNP) complexes, which have been shown to produce somatic mutations with high efficiency^32^. A dual-guide synthetic CRISPR RNA–dgRNP system has also been shown to enhance the efficiency of biallelic gene disruptions in zebrafish^32^. In this system, simultaneous injections of three distinct dgRNPs per gene into one-cell stage embryos dramatically improved the efficiency of biallelic gene disruptions with greater consistency than any single dgRNP injections^32^. Another method involves using a set of three synthetic gRNAs per gene, combining multilocus targeting with high mutagenesis at each locus^33^. Another method consistently converted >90% of injected embryos into biallelic knockouts using one or two optimized sgRNAs showed fully penetrant phenotypes and tested over 120 genes^34^.

## Precise genome editing using knock-in methods

One of the key applications of CRISPR–Cas9, aside from generating knockout alleles, is the creation of ‘knock-in’ alleles, where specific DNA sequences are inserted into the genome at targeted locations. One common approach for CRISPR knock-in in zebrafish involves using single-stranded oligodeoxynucleotides (ssODNs) as donor templates. These ssODNs contain the desired knock-in sequence flanked by homology arms that match the target genomic region. When co-injected with the CRISPR–Cas9 system, the ssODN can integrate into the genome through HDR^35^. Using this approach, Carrington et al. performed targeted insertion of epitope tags, as well as single nucleotide mutations, to recapitulate pathogenic human mutations^36^. They also developed a fluorescent PCR-based method to screen for knock-in alleles, which is rapid and inexpensive. Another approach utilizes long single-stranded DNA (lssDNA) as a donor template. This method has been shown to efficiently knock-in sequences encoding composite tags into the zebrafish genome by using a combination of CRISPR–Cas9 ribonucleoprotein (RNP) complex and lssDNA to achieve efficient knock-in of an approximately 200 base-pair sequence^37^.

The efficiency of CRISPR knock-in in zebrafish can vary depending on factors such as the target locus, the length and complexity of the knock-in sequence and the delivery method^38^. While knock-in efficiencies can be relatively low, recent studies have reported improved efficiencies through optimization strategies such as using antisense asymmetric oligonucleotides with longer homology arms^38^. One crucial insight is the strong inverse relationship between knock-in efficiency and the distance of the modification to the cut site. This highlights the importance of careful guide RNA design to maximize knock-in efficiency^35^. Like zebrafish, CRISPR knock-ins in mice can be achieved using ssODNs or plasmid donor templates with homology arms^39^. These templates are co-injected with the CRISPR–Cas9 system into mouse zygotes. Several factors influence the efficiency of CRISPR knock-in in mice, including the selection of sgRNA, the type of donor DNA template and the delivery method^39^.

One area of caution in generating knock-ins is that unexpected changes can occur and errors are frequently incorporated near the area of insertion. There is no community standard for demonstrating the broader locus surrounding the insertion site has not been altered and there have been documented cases of larger chromatin deletions in mice caused by CRISPR–Cas9 cleavage^40^ and clear genomic alterations in zebrafish, including the deletion of the distal telomeric region downstream of a knock-in at the *npas4l* locus^41^, and the commonly used ‘GeneWeld’ technology^42^ shows fairly high frequencies of integration errors at both the 5′ and 3′ junctions.

## Expansion of double-stranded cutting enzyme target sites

*S.* *pyogenes* Cas9 (SpCas9) is the most widely used and efficient Cas9. While the Cas9 is highly efficient, its targeting range is restricted by its protospacer adjacent motif (PAM) sequence (NGG). This restricts the ability of Cas9 to edit to roughly 1 in 16 genomic sites. While SpCas9 can sometimes tolerate slightly different PAMs (NAG or NGA), many potential targets, especially in gene-rich regions with high AT content, remain out of reach. To overcome this PAM restriction, many groups are developing two main strategies: (1) identifying new orthologs with novel PAM sequences and (2) engineering SpCas9 to create variants that can either recognize different PAM sequences or that can function without needing a PAM, thereby greatly expanding the scope of gene editing.

Over the past 10 years, researchers have characterized multiple Cas9 orthologs from different species. These orthologs recognize different PAM sequences, expanding the targeting range. Each ortholog has unique features such as size, PAMs and editing efficiencies. For example, *Staphylococcus aureus* Cas9 (SaCas9, NNGRRT PAM) and *Campylobacter jejuni* Cas9 (CjCas9, N4RYAC PAM), are smaller in size and therefore suitable for in vivo delivery using viral vectors such as adeno-associated virus (AAV), where packaging size is limited^43^. In addition to the Cas9 enzymes, researchers have explored other CRISPR–Cas systems, including type V Cas12 systems, which offer some distinct advantages. The Cas12 enzymes, such as Cas12a (Cpf1), recognize T-rich PAM sequences (for example, TTTV) and produce staggered DNA cuts, unlike the blunt DSBs of Cas9, facilitating precise gene insertion via HDR^44,45^. In addition, Cas12 systems process their own crRNA arrays, enabling multiplexed gene editing with a single transcript. Other type V effectors such as Cas12b and Cas12f have also been characterized. Cas12b is heat tolerant and adapted for mammalian editing, while the ultracompact Cas12f (Cas14) is ideal for AAV delivery, though its efficiency is still being improved. These systems expand the CRISPR toolbox with distinct PAM requirements, cleavage patterns and versatile applications. Various Cas9 orthologs and Cas12 enzymes, together with their PAM recognition sequences, are listed in Table 1. It is somewhat interesting to note that the ‘original’ spCas9 still seems to be the version that is the most efficient enzymatically across a very broad spectrum of conditions. A potential reduction in efficiency using a different version should be taken into account whenever designing an editing strategy.

**Table 1:** List of Cas9 variants and orthologous Cas enzymes.

Cas9 variant	Source/mutations	PAM sequence	Reference
SpCas9 (wild type)	*Streptococcus pyrogens*	NGG	^10^
SpCas9-NG	R1335V/L1111R/D1135V/G1218R/E1219F/A1322R/R1333P/R1335Q	NG (reduced activity at non-NGG)	^160^
VQR-SpCas9	D1135V/R1335Q/T1337R	NGAN > NGNG > NGCG	^161^
VRER-SpCas9	D1135V/G1218R/R1335E/T1337R	NGCG	^161^
xCas9	A262T/R324L/S409I/E480K/E543D/M694I/E1219V	NGN (broad recognition)	^162^
SpG-Cas9	D1135L/S1136W/G1218K/E1219Q/R1335Q/T1337R	NGN (prefers NGG/NGA/NGT)	^163^
SpRY-Cas9	D1135L/S1136W/G1218K/E1219Q/R1335Q/T1337R/T1642I/Y1669A	NRN (R = A or G)	^163^
SpCas9-NRRH	D1135V/R1335P/T1337R/T1642R/A1722R/S1738R	NRRH (R = A or G; H = A, C, or T)	^164^
SpCas9-NRTH	D1135N/R1335P/T1337R/T1642R/A1722R/S1738R	NRTH (R = A or G; H = A, C, or T)	^164^
SpCas9-VRQR	D1135V/R1335Q/T1337R/D1356R	NGA	^161^
SpCas9-NAG	D1135E	NAG	^165^
SaCas9	*Staphylococcus aureus*	NNGRRT	^166^
SaCas9-KKH	*Staphylococcus aureus*	NNNRRT	^161^
ScCas9	*Streptococcus canis*	NNG	^167^
FnCas9	*Francisella novicida*	YG (Y = C or T)	^168^
St1Cas9	*Streptococcus thermophilus*	NNAGAAW	^169^
NmeCas9	*Neisseria meningitidis*	NNNNGATT	^170^
Nme2Cas9	*Neisseria meningitidis*	NNNNCC	^171^
eNme2Cas9	*Neisseria meningitidis* (evolved)	NNNNTN or NNNNCN	^172^
CjCas9	*Campylobacter jejuni*	NNNNRYAC	^43^
GeoCas9	*Geobacillus stearothermophilus*	NNNNCRAA	^173^
SauriCas9	*Staphylococcus auricularis*	NNGG	^174^
iSpymac-Cas9	*Streptococcus macacae* and *Streptococcus pyogens chimeric*	NAAN	^175^
LbaCas12a	*Lachnospiraceae bacterium*	5′-TTTN	^45^
AsCas12a (Cpf1)	*Acidaminococcus* sp.	5′-TTTV (V = A, C, or G)	^45^
AsCpf-RR and AsCpf-RVR	*Acidaminococcus* sp. S542R/K607R and S542R/K548V/N552R	5′-TYCV, 5′-TATV (V = A, C, or G; Y = C or T)	^176^
enAsCas12a	*Acidaminococcus* sp.	5′-TTN	^177^
FnCas12a	*Francisella novicida*	5′-TTN	^45^
AaCas12b	*Alicyclobacillus acidoterrestris*	5′-TTTN	^178^
DpbCas12e (CasX)	Deltaproteobacteria	5′-TTCN	^179^
Un1Cas12f1	Uncultured organism	5′-TTTR (R = A or G)	^44^

## CBEs

Most genetic variants are single-nucleotide variants. For example, pathogenic point mutations in humans predominantly involve CG-to-TA conversions and account for nearly half of all mutations, while AT-to-GC changes represent about 14% (ref. ^46^) of the variants. Previously, directly testing variants in vivo required HDR-mediated knock-in approaches, which are orders of magnitude lower efficiency than NHEJ-mediated knockouts. This is because, in vertebrates, the NHEJ repair mechanism is preferred during DNA repair, resulting in low utilization of the HDR pathway. To address these challenges, innovative CRISPR technologies using various strategies have been developed^46–48^.

In 2016, the first ‘base editor’, a cytosine base editor (CBE), was developed that converted cytosine (C) to uracil (U), which is then repaired to thymine (T) during DNA replication, resulting in a C-to-T (or G-to-A opposite strand) substitution. The first-generation CRISPR base editor (BE1) consisted of a deactivated Cas9 protein (dCas9) fused to a cytidine deaminase enzyme. This enzyme, derived from the rat *APOBEC1* gene, is responsible for the C-to-U conversion^48^. The second-generation base editor (BE2) incorporated an uracil glycosylase inhibitor (UGI) to prevent the removal of uracil during DNA repair, which improves editing efficiency compared with BE1 (ref. ^48^). To further enhance efficiency, the third-generation base editor (BE3) was developed, which restores half of the catalytic activity of dCas9 to make it ‘nickase’, which cuts one strand of the DNA^48^. Base editing technology continued to be refined and many other variants were developed, such as BE4, BE4max and BE4-Gam, to include additional UGI copies and with optimized linker length to improve flexibility and reduce unintended edits^49^. By incorporating the Gam protein, which binds to DNA double-strand breaks, BE4 further reduced unwanted indels. The more improved and widely used CBEs, such as AncBE4max, include codon optimizations and enhanced nuclear localization signals, leading to significantly improved efficiency^50^. Nishida et al. developed another CBE called Target-activation-induced cytidine deaminase (AID), which incorporates nCas9 and Petromyzon marinus cytidine deaminase 1 (pmCDA1), a deaminase structurally and functionally like rAPOBEC1 (ref. ^51^). Target-AID is an efficient base editor, but it generates random changes in a small window, so it is useful for broadly testing sequence changes in the target region of a gene but less useful for individuals who need to generate a specific variant.

Given that APOBEC1-based CBEs primarily edit cytosines in the TC sequence context, Liu’s group utilized phage-assisted continuous evolution to create improved CBEs. They developed evoAPOBEC1-BE4max, which efficiently edits cytosines in the G/C sequence context, and evoFERNY-BE4max, a variant with a 30% smaller deaminase^52^. Further optimization to base editors focused on narrowing the editing window and improving editing precision to reduce bystander mutations. This has been achieved by modifying the deaminase component of the editors. Variants such as YE1-BE3 and EE-BE3 have successfully minimized off-target base conversions^53^. In addition, swapping the rat APOBEC1 enzyme in CBEs with engineered human APOBEC3A (A3A) has shown promise for precise editing^54^.

A primary limitation in base editing is the restricted availability of CRISPR target sites owing to dependencies on the PAM and the position of target nucleotide within the editing window, which is 4–8 nt of the protospacer sequence (upstream of PAM). To overcome these constraints, researchers have developed base editors using alternative Cas enzymes and Cas9 orthologs or variants, as described above, further expanding the targeting range.

## ABEs

Similar to CBE, adenine base editors (ABEs) were developed in 2017. ABEs convert AT base pairs to GC without inducing DSBs^46^. ABEs achieve high editing efficacy with minimal off-target effects compared with earlier editors such as BE3. The ABE system integrated nCas9 with an engineered adenine deaminase, which converts A to inosine, read as G by DNA polymerase, enabling AT-to-GC base conversions. ABE7.10, the first widely used ABE, achieved 58% editing efficiency with 99% product purity and less than 0.1% off-target activity, making it a precise and efficient editor^46^. Subsequently, ABE8e was developed through phage-assisted evolution, demonstrating a 590-fold increase in activity compared with ABE7.10 and enhanced compatibility with various Cas homologs^55^. Similarly, ABE8s, created through directed evolution, exhibit higher editing efficiency, particularly at challenging loci^56^. To address bystander mutations, NG-ABE9e was engineered, reducing off-target editing by up to sevenfold while maintaining high efficiency^57^. Specific mutations in ABEs can modulate their behavior, such as the D108Q mutation reducing cytosine deamination activity and the P48R mutation creating a TC-specific base editing tool^58^. By structure-guided engineering, ABE9 was generated with a limited editing window of one to two nucleotides to minimize bystander mutations. It has low RNA off-target activity and has no detectable Cas9-independent DNA off-target activity, thus making it more specific^59^. Furthermore, to reduce cytosine bystander mutations, ABE10 was designed using artificial intelligence-based methods. ABE10 is an improved adenine deaminase enzyme that has reduced sequence identity (65. 3%) with the previous versions of the enzyme and has been shown to have higher efficiency and reduced off-target activities compared with ABE8 (ref. ^60^).

The gene TadA had higher specificity, smaller size and lower Cas-independent DNA and RNA off-target editing activity than the APOBEC1-based cytosine deaminases, so the TadA enzyme was further evolved to generate TadA-derived CBEs (TadCBEs) that enable programmable C•G-to-T•A transitions while maintaining high specificity^61–64^. These editors were developed by evolving the adenosine deaminase TadA to perform cytidine deamination, resulting in enzymes with altered selectivity favoring deoxycytidine over deoxyadenosine^61^. TadCBEs offer comparable or superior on-target activity and significantly reduced off-target editing compared with traditional CBEs^61,63^. Last, innovative ‘dual-function’ editors, such as A&C-BEmax and SPACE, combine the functionalities of CBEs and ABEs in a single enzyme, enabling simultaneous cytosine-to-thymine and adenine-to-guanine edits^65,66^. Although many tools have not yet been widely tested in animal models, they hold great potential for expanding genomic manipulation capabilities. A number of prominent cytosine and adenine base editors are listed in Table 2.

**Table 2:** List of base editors.

Base editor	Deaminase enzyme	Cas enzyme	Editing window (nt)	Reference
**CBEs**				
BE3	rAPOBEC1	nCas9-H840A	4–8	^48^
BE4max	rAPOBEC1	nCas9-D10A	4–8	^180^
AncBE4max	Ancestral APOBEC1	nCas9-D10A	4–8	^180^
SaBE4	rAPOBEC1	nSaCas9-D10A	3–12	^49^
BE3-Gam	rAPOBEC1	nCas9-D10A	4–8	^49^
SaBE3-Gam	rAPOBEC1	nSaCas9-D10A	3–12	^49^
BE4-Gam	rAPOBEC1	nCas9-D10A	4–8	^49^
SaBE4-Gam	rAPOBEC1	nSaCas9-D10A	3–12	^49^
CDA1-BE3	PmCDA1	nCas9-D10A	4–8	^49^
AID-BE3	hAID	nCas9-D10A	4–8	^49^
evoAPOBEC1-BE4max	rAPOBEC1	nCas9(D10A	3–8	^52^
evoFERNY-BE4max	FERNY*	nCas9-D10A	1–8	^52^
evoCDA1-BE4max	PmCDA1	nCas9-D10A	1–13	^52^
SaBE3	rAPOBEC1	nSaCas9-H840A	3–12	^53^
SaKKH-BE3	rAPOBEC1	nSaKKHCas9-H840A	3–12	^53^
YEE-BE3	rAPOBEC1-YEE	nCas9-H840A	5–6	^53^
EE-BE3	rAPOBEC1-EE	nCas9-H840A	5–6	^53^
Target-AID	PmCDA1	dCas9- D10A, H840A	3–7	^51^
Target-AID-NG	PmCDA1	nCas9-D10A-NG	2–8	^160^
AIDx	hAIDx	dCas9-D10A, H840A	5–9	^181^
dCas9-AIDx	hAIDx	dCas9- D10A, H840A	5–9	^181^
HF-BE3	rAPOBEC1	HF1-nCas9-H840A	4–8	^67^
xBE3	rAPOBEC1	nxCas9-D10A	4–8	^162^
BE-PLUS	rAPOBEC11-scFV	nCas9-D10A	4–16	^182^
iSpy-macnCas9-BE3	rAPOBEC1	iSpy-macnCas9-D10A	4–8	^175^
R33A-BE4	rAPOBEC1-R33A	nCas9-D10A	5–7	^88^
R33A/K34A-BE4	rAPOBEC1-R33A/K34A	nCas9-D10A	5–6	^88^
BE3- R33A	rAPOBEC1-R33A	nCas9-D10A	5–7	^183^
BE3-R33A/K34A	rAPOBEC1-R33A/K34A	nCas9-D10A	5–6	^183^
eA3A-BE3	Evolved hAPOBEC3G-N57G	nCas9-D10A	4–8	^54^
hA3A-BE3	Evolved hAPOBEC3A	nCas9-D10A	2–13	^184^
SpRY CBE4max	rAPOBEC1	SpRY-nCas9-D10A	3–9	^163^
SpG CBE4max	rAPOBEC1	SpG-nCas9-D10A	3–9	^163^
nCDA1-BE3	PmCDA1Δ	nCas9-D10A	3–4	^185^
Tad-CBE	Evolved TadA	nCas9-D10A	3–8	^61^
Td-CBEs	Evolved TadA	nCas9-D10A	6–7	^62^
eTd-CBE	Evolved TadA	nCas9-D10A	5–6	^62^
zevoCDA1-BE4max	Evolved CDA1	nCas9-D10A	1–9	^75^
zevoCDA1-NL	Evolved CDA1	nCas9-D10A	1–7	^75^
zevoCDA1-198	Evolved CDA1	nCas9-D10A	1–5	^75^
zevoCDA1-SpRY-BE4max	Evolved CDA1	SpRY-nCas9-D10A	1–9	^75^
**Adenine base editors**				
ABE7.10	TadA-TadA*	nCas9 (D10A)	4–7	^46^
xABE	TadA-TadA*	x-nCas9 (D10A)	4–7	^162^
ABEmax	TadA-TadA*	nCas9 (D10A)	4–7	^50^
NG-ABEmax	TadA-TadA*	nCas9-NG (D10A)	4–8	^160^
ABEmaxAW	TadA-E59A-TadA*-V106W	nCas9 (D10A)	4–8	^67^
VQR-ABEmax	TadA-TadA*	VQR-nCas9 (D10A)	4–8	^186^
VRER-ABEmax	TadA-TadA*	VRER-nCas9 (D10A)	4–8	^186^
VRQR-ABEmax	TadA-TadA*	VRQR-nCas9 (D10A)	4–8	^186^
Sa-ABEmax	TadA-TadA*	Sa-nCas9 (D10A)	4–14	^186^
SaKKH-ABEmax	TadA-TadA*	SaKKH-nCas9 (D10A)	4–14	^186^
miniABEmax	TadA*	nCas9 (D10A)	4–7	^186^
ABE7.10 F148A	TadA-F148A-TadA*-F148A	nCas9 (D10A)	5	^187^
ScCas9-ABE (7.10)	TadA-TadA*	Sc-nCas9 (D10A)	4–7	^188^
SECURE-ABEs	TadA*-K20A/R21A or TadA*-V82G	nCas9 (D10A)	4–7	^183^
ABE8e	TadA*	nCas9 (D10A)	4–7	^55^
ABE8s	TadA*	nCas9 (D10A)	3–10	^56^
ABE9	TadA*	nCas9 (D10A)	5–6	^59^
ABE10	Artificial intelligence-designed TadA	nCas9 (D10A)	5–7	^60^
ABE-Ultramax	TadA*	nCas9 (D10A)	3–9	^74^
ABE-Umax-ex1	TadA*	nCas9 (D10A)	4–12	^74^
ABE-Umax-ex2	TadA*	nCas9 (D10A)	8–16	^74^
ABE-Umax-rest1	TadA*	nCas9 (D10A)	5–6	^74^
ABE-Umax-ex1-rest1	TadA*	nCas9 (D10A)	4–6	^74^
ABE-Umax-ex2-rest1	TadA*	nCas9 (D10A)	12–15	^74^

## Base editing in vivo

Over the past 8 years, several base editing tools have been developed, but their adoption in vivo such as in zebrafish research has been relatively slow. The first application of a CBE in zebrafish was BE3; two independent groups achieved 9–25% C→T editing by microinjecting mRNA or RNP complexes^67,68^. Zhang et al. utilized base editing utilizing Target-AID editor to establish a disease model for oculocutaneous albinism^69^. The AncBE4max variant demonstrated a threefold increase in efficiency compared with BE3 in zebrafish^70^. In another study, BE4-Gam and AncBE4max were employed to model oncogenic mutations in the tumor suppressor gene *tp53*, showcasing their utility in cancer modeling^71^. Furthermore, BE4max was fused with PAM-less Cas9 (SpRY) to create a variant that eliminated the NGG PAM restriction, achieving exceptionally high editing activity of up to 85% (ref. ^72^). In addition, Cornean et al. developed ACEofBASEs, an online platform for efficient sgRNA design for base editing, and employed AncBE4max and EvoBE4max editors to introduce precise mutations in zebrafish and medaka, including stop-gain and missense variants in genes such as *oca2* (eye pigmentation) and *kcnh6a* (potassium channels)^73^. Recently, Qin et al. introduced ABE-Ultramax (ABE-Umax), a novel adenine base editor developed through zebrafish in vivo screening, achieving up to 90% editing efficiency—three times higher than ABE8e. They also developed a suite of editors with expanded editing windows and precision tools to minimize bystander mutations^74^. These advances were demonstrated by creating multiple zebrafish disease models, including missense and splicing mutations, enabling precise functional validation of single-nucleotide variants^74^. Similarly, Zhang et al. optimized zevoCDA1 and its variant, zevoCDA1-198, to enhance C-to-T editing efficiency, relax PAM restrictions, narrow the editing window and reduce off-target effects in zebrafish^75^. In *Xenopus laevis* embryos, BE3 RNP was successfully used for C-to-T editing in the *Tyr* and *Tp53* genes, achieving editing efficiencies of 20% and 5%, respectively. In addition, BE3 induces approximately 20% indels in *Xenopus*, somewhat limiting its utility^76^.

The first mouse models were generated using base editing targeting the *Dmd* and *Tyr* loci to introduce a premature stop codon provided by C-to-T base editing within a coding exon. To this end, BE3 mRNA and guide RNAs targeting the Dmd gene were injected into mouse embryos. More than 50% of the founder mice carried C-to-T mutations; however, other off-target mutations, including deletions, were also present. Other reports also demonstrated off-target indels associated with CBE-based editors^77^. To target the *Tyr* gene, BE3 protein and sgRNA were delivered as RNP complex by electroporating mouse zygotes, and two homozygous founders were obtained carrying the desired mutation in the *Tyr* gene. Sasaguri et al. compared the editing efficiency of BE3 and Target-AID using the mRNA and sgRNAs targeting the presenilin-1(*psen-1*) gene in mouse zygotes. BE3 consistently demonstrated higher base-editing efficiency in a range of 10–60% compared with Target-AID, which was 3–30%, but it also generated a higher frequency of indels^78^. The high efficiency of CRISPR–Cas9 enables the editing of multiple genes simultaneously, Zhang et al. tested whether a similar approach was possible using CBE by simultaneously targeting the *vGlut3*, *Otof* and *Prestin* genes^79^. This introduced C-to-T transitions that resulted in premature termination codons (CRISPR-stop). This method efficiently generated homozygous F0 mouse mutants for single or triple genes, bypassing the labor-intensive process of mouse breeding. This represents a significant step forward for functional genomics in vivo.

Over the past few years, base editing has been successfully applied in mouse models to address various genetic diseases; for example, spinal muscular atrophy is caused by mutations in the gene *SMN1*, resulting in a deficiency of the SMN protein essential for the survival of motor neurons. Arbab et al. used adenine base editing to convert a specific T•A base pair in the *SMN2* to a C•G base pair. This conversion effectively transforms *SMN2* into a functional copy of *SMN1*, restoring SMN protein levels and leading to significant improvements in motor function in treated mice^80^.

Amyotrophic lateral sclerosis (ALS) is a disease characterized by the degeneration of motor neurons. Approximately 20% of all inherited cases of ALS are caused by mutations within the *SOD1* gene. Lim et al. used a split-intein CBE to disrupt mutant SOD1 expression in an ALS mouse model, resulting in reduced expression of mutant SOD1, improving neuromuscular function and extending survival^81^. Split-intein CBE involves splitting the Cas9 protein or other base editor components into two parts fused to inteins. These parts reconstitute into a functional base editor only when codelivered and expressed in the same cell. This design enables spatial or temporal control over activation and simplifies the delivery of large constructs, especially using vectors such as AAVs.

Progeria, or Hutchinson–Gilford progeria syndrome, is a very rare disease characterized by early onset accelerated aging caused by a mutation in the *LMNA* gene, which leads to the production of a toxic protein called progerin. Koblan et al. used an adenine base editor in a mouse model of progeria to correct a dominant negative mutation CG-to-TA mutation (c.1824C > T; p.G608G) in LMNA. The treated mice displayed lower progerin levels than saline-injected control mice with progeria^82^. This ultimately resulted in significantly improved tissue health and the treated mice lived two and a half times longer than the control mice, reaching an age equivalent to the beginnings of old age in healthy mice.

Base editing has been successful in various mouse models of genetic diseases, paving the way for its clinical application. Currently, it is being tested as a therapeutic approach for treating genetic disorders in several preclinical studies^83^. Success in these preclinical experiments has led to the initiation of clinical trials to explore the use of base editing therapies in humans.

## Genome editing using prime editing

**Fig. 3:**
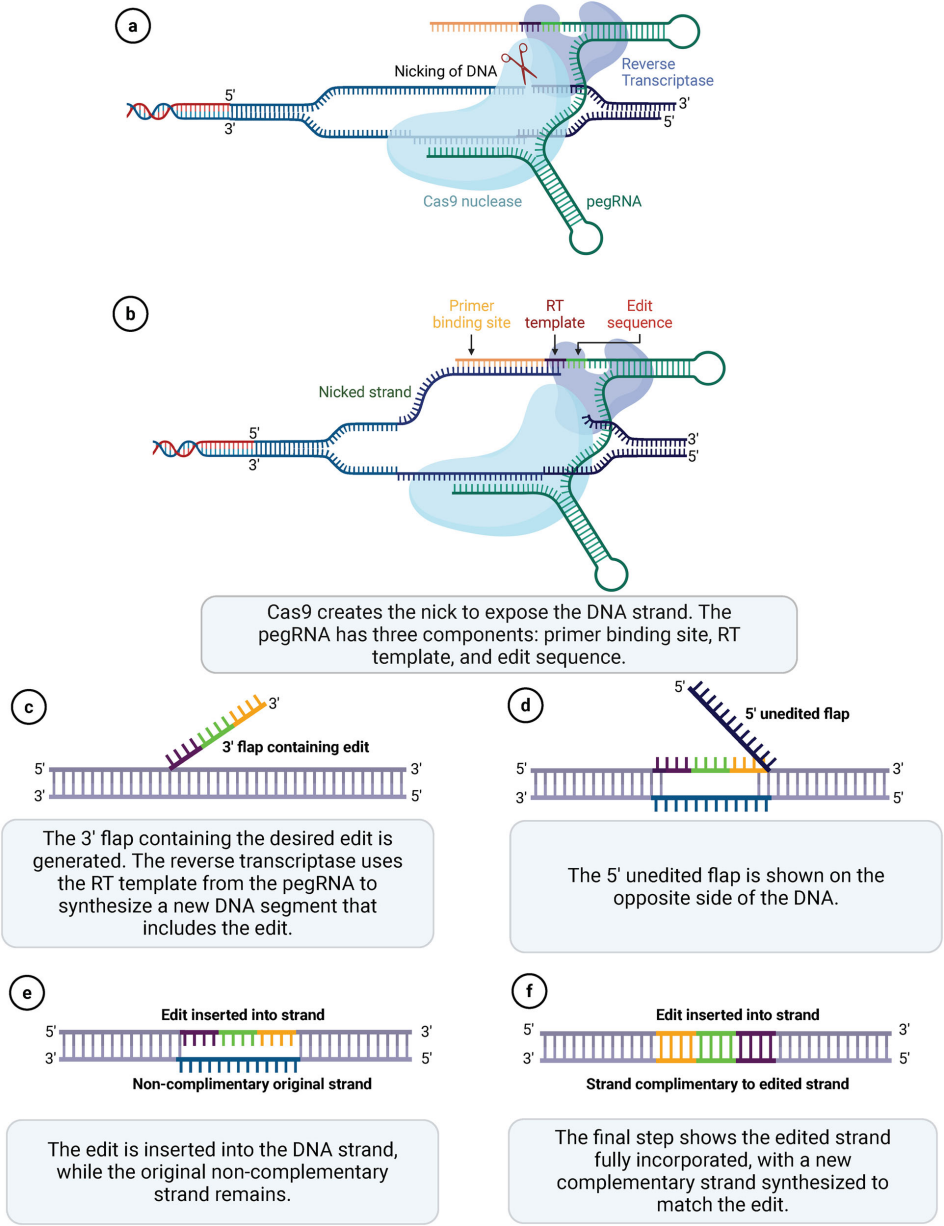
Mechanism of CRISPR prime editing for precise genome modification. **a** Prime editing begins with Cas9 nickase fused to a RT and guided by a pegRNA. Cas9 nicks the target DNA strand, allowing RT to access the DNA for editing. **b** The pegRNA contains three components: a PBS, a RT template and an intended edit sequence. The nicked DNA strand anneals to the PBS, allowing reverse transcription of the edit. **c** A 3′ flap containing the desired edit is synthesized from the RT template, generating a new DNA segment with the desired sequence. **d** A competing 5′ unedited flap forms from the original DNA strand. The resolution of this heteroduplex determines which sequence is retained. **e** The edited strand is integrated into the genome, displacing the original noncomplementary strand. **f** DNA repair mechanisms synthesize the complementary strand to match the edited sequence, completing the prime editing process with a permanent and precise edit. (Figure created using Biorender.com).

Prime editing allows for targeted insertions, deletions and all 12 possible base-to-base conversions^84^. This ‘search-and-replace’ genome-editing technology functions without requiring DSBs or donor DNA templates, a significant advantage that sets it far apart from traditional CRISPR–Cas9 systems and base editors, which have limited editing scope and higher potential for off-target effects^84^. A prime editor comprises a modified Cas9 enzyme and an engineered Moloney murine leukemia virus reverse transcriptase (MMLV RT) guided by a prime editing guide RNA (pegRNA) (Fig. 3). The pegRNA targets the specific DNA site and carries the desired edit within its long tail sequence. The PE system works through a ‘search-and-replace’ mechanism. First, the pegRNA guides the Cas fusion protein to the target DNA site, where the nCas9 nickase creates a single-strand nick. The primer binding site (PBS) of the pegRNA then anneals to the nicked DNA strand and the RT enzyme uses the 3′ extension of the pegRNA as a template to synthesize the edited DNA sequence. This newly synthesized DNA strand is then incorporated into the genome through the cellular DNA repair machinery, resulting in the desired modification. The prime editing system has evolved through several generations to improve efficiency and reduce unintended byproducts. PE1, the first-generation prime editor, combines a Cas9 H840A nickase with the wild-type MMLV RT but demonstrates modest editing efficiency, converting less than 5% of targeted alleles^84^. PE2 addressed these limitations by introducing five mutations to the MMLV RT domain, enhancing its thermostability, processivity and binding affinity, which resulted in a 1.6–5.1-fold increase in editing efficiency^84^. PE3 further advanced the system by incorporating an additional sgRNA to nick the nonedited DNA strand, improving strand replacement and editing efficiency, albeit with a slightly increased risk of indel formation. To mitigate this risk, PE3b introduced a complementary-strand nick specific to the edited sequence, reducing indels without sacrificing efficiency^84^. Subsequent iterations, PE4 and PE5, aimed to inhibit DNA mismatch repair by targeting MLH1, with PE4 based on PE2 and PE5 on PE3, utilizing a dominant-negative MLH1 protein to enhance editing outcomes^85^. Other innovations, such as PEmax^85^, an improved version of PE4 and PE5 variants, and ePPE, a variant that was developed by removing the RNase H domain in the RT enzyme and replacing it with viral nucleocapsid protein^86^, focused on improving nuclear import, tethering peptides and optimizing the RT domain for greater efficiency. Enhancements to the prime editor protein involve engineering RT with mutations such as the pentamutant RT to increase efficiency^87^, developing smaller editors such as PE2ΔRnH through phage-assisted evolution, called PE6 (ref. ^88^), and creating an intein-split construct for AAV-mediated delivery^89^. Recently, a new PE system, PE7, has been enhanced by incorporating the RNA-binding protein La, which strengthens the interaction between the prime editor and the pegRNA^90^. This improved interaction is essential for efficient editing. Compared with its predecessors, PE7 shows superior editing performance, especially when using engineered pegRNAs (epegRNAs) and synthetic pegRNAs optimized for La binding.

Prime editing has undergone numerous modifications to enhance its efficiency and expand its capabilities, focusing on improving the prime editor protein, the pegRNA and developing new strategies. For pegRNA, modifications include refolding and introducing mutations to reduce autoinhibitory activity^91^, integrating structured RNA motifs such as xrRNA for improved stability^92^ and optimizing primer binding site and reverse transcription template length to balance efficiency and accuracy^91^, systematically analyzing these parameters in zebrafish and human cells to improve editing rates and minimize off-target effects^91^. Other advancements in pegRNA include modified versions such as apegRNA and spegRNA, developed by improving pegRNA secondary structure and introducing same sense mutations, increasing indel frequency without significantly affecting unexpected indels or byproducts^93^. epegRNAs integrate structured RNA motifs into the 3′ end of pegRNAs, increasing editing efficiency in various cells by three to four times without increasing off-targeted activity^94^.

Approaches such as prime editing-Cas9-based deletion and repair leverage nucleases (not nCas9) that create DSBs in combination with RT and a pair of guide RNAs (pegRNAs), enabling the precise deletion of genetic sequences exceeding 10 kb and the insertion of up to 60 bp into cellular DNA. In a mouse model of tyrosinemia, prime editing-Cas9-based deletion and repair successfully removed a 1.38 kb pathogenic insertion within the *Fah* gene. This precise editing restored the functionality of the target gene by accurately repairing the missing genetic connection, leading to the renewed expression of the *Fah* gene in the liver. PRIME-Del uses a pair of pegRNAs to accurately edit targets by deleting sequences up to 10 kb with higher accuracy (1–30%)^95^. GRAND, which also uses a pair of pegRNAs, has a higher editing efficiency of 63.0% for 150 bp inserts but can introduce byproducts and has a reduced efficiency of 28.4% for 250 bp insertions^96^. Bi-PE increases efficiency by 16 times and accuracy by 60 times by including a nick sgRNA near the pegRNA template sequence. This system can delete large DNA sequences and introduce small fragments into the deletion sites^97^. HOPE uses two pegRNAs with homologous 3′ terminals to target DNA double strands, balancing efficiency and accuracy^98^. TwinPE utilizes a prime editor protein and two pegRNAs to edit genes up to thousands of base pairs long^47^. ePE optimizes the pegRNA skeleton to improve point mutation editing efficiency by 1.9 times compared with standard prime editing^99^. Since most prime editors utilize SpCas9, which recognizes NGG PAM, thereby limiting the targeting sites, efforts to expand this limitation, utilizing Cas9 variants with alternate PAM recognition, have led to the development of various PE2 variants, including PE2-VQR, PE2-VRQR, PE2-VRER, PE2-NG, PE2-SpG and PE2-SpRY, which can target different PAM sequences^100^.

Several studies have demonstrated the successful application of prime editing in model organisms for modeling genetic disorders and correcting disease-causing mutations. For instance, prime editing was used to introduce a single-base deletion in the *Crygc* gene in mice to model a cataract disorder by targeting a single-base deletion, resulting in a high G-deletion rate (38.2%) and the development of a nuclear cataract phenotype in the mice^101^. In another study, prime editing was employed to correct a disease-causing mutation in the *Fah* gene in a mouse model of tyrosinemia, a metabolic liver disease^102^. Similarly, prime editing has been used to correct mutations in several conditions in mice such as Duchenne muscular dystrophy (*Dmd*)^103^, phenylketonuria (*Pah*)^104^, retinitis pigmentosa (*rpe65*)^102^ and the E342K mutation in the *SERPINA1* gene associated with alpha-1 antitrypsin deficiency^105^. Prime editing was used to perform single-base substitution within a transcription factor binding site (CArG box) in the mouse Tspan2 promoter^106^. The C > G substitution resulted in the near-total loss of *Tspan2* mRNA expression in the aorta and bladder tissues, rich in smooth muscles, while sparing heart and brain tissues.

### Polymerase-based prime editing

While effective, traditional prime editing has several drawbacks, including low efficiency in nondividing cells, error-prone RT leading to unintended mutations and limited processivity, which restricts the length of edits. To address these challenges, polymerase-based prime editing was developed by two independent groups^107,108^. This method replaces the RT with a DNA-dependent DNA polymerase and uses DNA instead of RNA as a template. This switch enhances gene editing by offering higher efficiency and accuracy, increased processivity for longer edits and better dNTP affinity, thus improving performance in nondividing cells. Another prime editing method, referred to as ‘click editing’, involves a tripartite protein complex consisting of an RNA-programmed DNA nickase, a DNA-dependent DNA polymerase and a single-stranded DNA tethering domain (histidine–hydrophobic-histidine (HUH) endonuclease)^107^. The DNA template, called click DNA (clkDNA), contains a primer binding site, a polymerase template with the desired edit and the HUH recognition site. One advantage of this system is the cost-effectiveness of the DNA template. Unlike the modified RNA–DNA hybrids used in other methods, the clkDNAs are standard DNA oligonucleotides that can be readily and cheaply synthesized. This makes click editing more accessible and affordable for research and therapeutic applications.

Prime editing represents a paradigm shift in genome engineering, combining the precision of base editors with the versatility of traditional CRISPR–Cas systems. Its ability to correct, insert or delete DNA sequences with high accuracy positions it as a critical tool for advancing genetic research and therapeutic development. While challenges remain in terms of efficiency, off-target effects and delivery, further optimization efforts are required for broader applications of prime editing in various fields, including disease modeling, gene therapy and synthetic biology.

## Transcriptional regulation using CRISPR–Cas

### CRISPR interference

As described above, the majority of research efforts using CRISPR–Cas systems has been on genome editing either through preserving the nuclease function of Cas proteins and causing single-strand breaks or DSBs, or through inactivating the nuclease activity and adding a separate editing function to the protein in one way or another. However, researchers have also realized there is tremendous value in being able to rapidly design a protein with a specific biding location anywhere in the genome. The most obvious utility of such a capability would be to make artificial transcription factors that could either activate or inhibit transcription of a target gene. Within a year of the first papers describing the nuclease activity of the CRISPR–Cas9 complex, researchers showed a catalytically dead Cas9 (dCas9) could bind close to the transcription start site and prevent transcription of genes in *E.* *coli*^109^. The system was called ‘CRISPR interference’ or ‘CRISPRi’ for short. Variants of Cas9 fused to known eukaryotic transcriptional repressors soon followed. Krüpple-associated box domains (KRAB domains), known transcriptional repressors used by over 400 human zinc-finger proteins^110^, were the first fusions made with dCas9. Since the initial tests using the KRAB domain from the KOX1 zinc-finger protein^111^, two other versions have emerged: KRAB-MeCP2 (ref. ^111^) and the ZIM3 KRAB domain^111^, both showing stronger inhibition than the original construct in mammalian cell culture.

Efficacy in cell culture often does not replicate in vivo, where genomic responses can be more complex. However, studies in whole animals are essential for understanding gene functions as tissues or organs can significantly modify the outcome of gene inactivation. Effective CRISPRi in vivo could potentially simplify conditional knockout transgenesis, as a single tissue-specific CRISPRi transgene could inactivate gene expression instead of the typical approach of relying on bringing two transgenics together, one expressing a recombinase tissue specifically and the second one containing the target gene flanked by recombination sites (for example, cre/lox systems). A CRISPRi system could be characterized in one generation (approximately 3 months for mice or zebrafish). At the same time, the traditional approach could take over a year in mice and potentially longer in zebrafish where conditional knockouts are still not routinely done. Several studies have shown that CRISPRi transgenes can effectively reduce gene expression in mice although in some cases, inducible forms of CRISPRi (for example, doxycycline) were more effective at maintaining gene inactivation than shutting off genes that had already been activated^112^.

### CRISPR activation

Similar efforts have been made to use dCas9 fusions as synthetic transcription factors that can activate gene expression, termed ‘CRISPR activation’ (CRISPRa). Most commonly the dCas9 is fused to the transcriptional activation domain VP64 or its various derivatives^113^. Other strategies include fusion to histone demethylases or acetylases, which change chromatin availability and can activate expression indirectly^114^. There have not been any in vivo examples of CRISPRa as single transgenes to activate gene expression as they do not present a particular advantage over traditional methods for ectopic gene expression in mice or zebrafish. Both the CRISPRi and CRISPRa technologies do run the risk of off-target effects, which are probably higher than off-target rates for Cas9 nuclease activity as the requirements for binding to the genome, including mismatch tolerance, are lower than they are for activating DNA cleavage.

## CRISPR-based epigenetic editing: targeting histone and DNA methylation

Biochemical modifications on both the genomic DNA and the histones packaging chromatin are known to regulate gene expression. Several studies have utilized chromatin modification enzymes fused to dCas9 as another mechanism to alter gene expression. One key modification is the methylation of the fourth lysine in the histone 3 protein (H3K4)^115^. K4 can be modified from one to three methylations; the addition of methylation can increase chromatin accessibility, thereby increasing gene expression, while removing methylation can cause the chromatin to become more compact, resulting in reduced transcriptional rates. Several studies have used different enzymes fused to CRISPR to initiate changes in H3K4 methylation, including LSD1 (reduces trimethyl), EZH2 (reduces trimethyl), PRDM9 (adds trimethyl) and SMYD3 (adds trimethyl)^116–119^. These approaches have been shown to decrease or increase gene expression in cell culture. These changes in expression level are rarely complete; more often, the expression changes are in the range of twofold up or down. In many cases, this might actually be a better outcome than fully inactivating or strongly overexpressing a gene of interest. For example, there are many cases where fully inactivating a gene is so disruptive as to cause complete cell or organismal death, while a hypomorphic mutation (or hypermorphic) results in a phenotype that reveals an essential role for that gene^120^. Related modifiers targeted other histone modifications, such as H3K27 methylation^121^, have also been developed with similar levels of success in cell culture.

In both eukaryotes and prokaryotes, DNA methylation at cytosine residues to form 5-methylcytosine is another known epigenetic mark that regulates gene expression^122^. Altering local methylation states represents another alternative method for altering gene expression, even after the proteins involved in editing the chromatin marks are no longer present in the cells. Fusing dCas9 to methyltransferase enzyme active domains such as DNMT3A^123^ or DNMT3A-DNMT3L^124^ resulted in targeted methylation of genomic DNA in many experiments. One group amplified the local activity of the DNMT3A enzyme by using a ‘SunTag’ strategy where multiple SunTag epitopes were fused to dCas9 and the DNMT3A had the single-chain variable fragment (scFv) interacting domain fused to the protein bringing multiple copies of DNMT3a to the targeted location^125^. Similar to the histone modifications, it was also difficult to control the potential off-target or too-broad local editing of methyltransferases. One group was able to improve specific targeting by fusing dCas9 to a fragment of the methyltransferase (M.SssI), which had its DNA-binding domain mutated to remove the natural affinity of M.SssI for binding DNA^126^. These alterations appeared to reduce off-target DNA methylation. To activate gene expression by modifying methylation, groups fused dCas9 to the TET1 protein. TET1 catalyzes the conversion of 5-methylcytosine to 5-hydroxymethyl cytosine. This conversion is purportedly the first step to DNA demethylation, which would ultimately lead to increases in transcription at the targeted locus. The resultant changes in gene regulation in all of these targeted methylation experiments were complex and indicative that we still do not fully understand the roles methylation plays in gene expression. Gene expression was typically reduced with the methyltransferases and increased with the TET1 fusions, but the degree of silencing or activation was challenging to predict as it varied considerably from locus to locus. As with the other forms of transcriptional regulation utilizing CRISPR–Cas, the chromatin mark approaches have been used a great deal in cell culture experiments, but the benefits of using them in animal studies have not yet been demonstrated.

## CRISPR as a tool for exploring higher-order chromatin organization

There has been increased interest in how the complex, higher order of chromatin organization might influence gene regulation. Both sequence-based^127,128^ and imaging-based^129,130^ experimental approaches have revealed genomic organization at tens to hundreds of thousands of base pairs that appear to regulate gene expression. Often termed ‘topologically associated domains’ or ‘TADs’, the interactions of these chromatin elements modulate gene expression in various contexts. Some of the earliest experiments first identified sequences that appeared to act as ‘insulators’, that is, regions of the chromatin that would block enhancers or silencers for other genes that were in close proximity to other genes that should not be controlled by these regulatory elements^131^. Eventually, researchers demonstrated that a major regulator of insulator sequences and other TAD structures were controlled by the DNA-binding protein CCCTC-binding factor (CTCF), which binds a defined sequence motif and mediates the creation of large DNA loop structures^132^. As our understanding of how the genome organized itself in 3D space improved, we began to recognize that it was potentially involved in sophisticated gene regulatory mechanisms and so it was, therefore, necessary to test function experimentally just as you would genes, enhancers and silencers.

Various strategies using CRISPR have been utilized to experimentally regulate higher-order chromatin. The most straightforward of these approaches was to ablate specific CTCF binding sites using the CRISPR–Cas nuclease activity to generate DSBs. These mutated sites would result in altered genomic topologies and changes in gene expression^133–135^. Another approach was for dCas9 to competitively bind to CTCF sites preventing formation of TADs^136^. One group used two heterologous dCas9 proteins (from *S.* *pyogenes* and *S.* *aureus*), with each protein fused to two protein domains, PYL1 and ABI1, that normally dimerize in the presence of plant phytohormone *S*-(+)abscisic acid^137^. The researchers then targeted one dCas9 to the b-globin promoter and the other construct to the locus control region (LCR) in K562 cells. They showed that the addition of *S*-(+)abscisic acid to the culture media triggered the expression of b-globin, and that expression was linked to bringing the locus control region and the b-globin promoter into close proximity. The published studies were all performed in cell culture, but stable disruptions of 3D chromatin structure in vivo are possible when targeting specific loci.

## High-throughput screening in vitro using CRISPR–Cas

So far, we have focused on using CRISPR–Cas9 at specific loci in the genome. While not every technique discussed has been demonstrated to be effective in vivo, it is reasonable to assume that any locus-specific engineering that works in cell culture can work in an animal if the conditions are optimized for the model organism being used. Scientists realized early on, however, that one of the powerful aspects of CRISPR–Cas systems is that the DNA-binding site can be re-engineered rapidly by swapping out the RNA guide sequence. It then becomes a straightforward computational problem to design a library of guide RNA sequences that could target the genome on a large scale.

Approximately a year after the RNA-guided nuclease function of CRISPR–Cas9 was described, the first global CRISPR–Cas9 mutagenesis experiments were performed. The first efforts targeted gene inactivation of all human coding genes via mutations caused by NHEJ^138^. Lentiviruses were made that contained both a sgRNA and the Cas9 coding sequences driven by independent promoters. Each viral particle contained a different sgRNA, and multiple sgRNA were designed for each gene in the human genome. Cells were infected at multiplicities of infection below one per cell to avoid multiple targets per cell. These first screens used positive selections (that is, loss of gene function led to survival under selection) such as drug resistance. After a selection period, the sgRNA regions of the vectors were amplified and sequenced, looking for enrichment of guides that would allow the cells to survive.

A second approach was to use negative selection for the screen. In the simplest case of negative selection, a screen for ‘essential’ genes in vitro, cells were infected with the CRISPR–Cas9 libraries and then grown out for a period of time. Any cells with essential genes inactivated would die off and their associated sgRNAs would be depleted from the sequenced amplicons^139^. The adoption of these screening approaches required bioinformatic approaches for analyzing the data and several approaches emerged that were adapted and improved versions of analyses used for the shRNA (RNAi) screens^140–142^. These approaches attempted to generate ‘gold standard’ lists of essential and nonessential genes and calculations that could account for variation in guide efficacy, which could then inform future screening efforts.

As the modified versions of CRISPR–Cas emerged, so did their use in genome-wide screening. CRISPRi was used in similar ways to inactivate gene expression^143,144^. Perhaps more interesting in this case is the use of CRISPRa in screening^143,144^. While there are several avenues for inactivating gene expression, the ability to overexpress genes is more unique and has the potential for making functional discoveries through a different modality. Similarly, while the focus of most research has been on targeting the coding regions of the genome, efforts have begun to target the much larger space of genomic regulatory elements, essentially enhancers and silencers scattered throughout the genome. Unlike coding regions, these regulatory sequences are generally more permissible to variation, the ‘rules’ for functionality are much more poorly understood and our ability to interpret phenotypes is much more challenging. Despite the challenges, researchers are beginning to target regions of the genome that are ‘conserved noncoding elements’ at scale^145^. In one study, the researchers targeted 10,674 genes and 26,385 conserved regions in 2227 enhancers in human neural stem cells and found that overall, the targeted enhancers had weaker effects on gene expression, as one would predict, but many enhancers severely disrupted stem cell self-renewal^145^. Targeting regulatory regions will be an essential component to studying genes in vivo where differential expression depending on context will be more critical than it is in vitro.

## High-throughput screening in vivo using CRISPR–Cas

While very powerful, screens performed in cell culture are fundamentally limited in the questions that can be asked. To capture the true complexities of organismal biology, it is necessary to perform experiments in vivo (or, to a lesser extent, in cultured organoids) where the interactions between different cell types, tissues or organs can be interrogated. Naturally, screenings in animals (or plants) will inherently have much lower throughput as both the delivery of CRISPR–Cas complexes and the downstream analysis of phenotypes are significantly more complicated than they are in cell culture. Therefore, researchers must either find a way to reduce the total number of targets screened or streamline and automate phenotyping to minimize post-treatment processing time. In addition, building a stable library of animals containing transgenes constitutively expressing sgRNAs ubiquitously once would allow researchers to repeatedly screen using different Cas protein drivers.

In zebrafish, an approach named ‘Multiplexed Intermixed CRISPR Droplets’ (‘MIC-Drop’), is an exciting new approach for high-throughput CRISPR–Cas in vivo screening^146^. The concept is relatively simple, sgRNAs targeting specific genomic loci are synthesized in 96-well format, one genomic locus per well. Then, Cas9 protein and a barcode sequence are added to each well, and the individual wells from the plate are then moved to a microdroplet maker (for example, a Bio-Rad QX200) and nanoliter-sized droplets are suspended in oil, one target per droplet. The droplet mixture can then go into an injection needle and one droplet can be injected into a single embryo. The embryos are screened for phenotypes at the appropriate age and then the barcodes of fish with a positive signal are amplified and sequenced to identify the locus of interest. The throughput for such a technique is in the hundreds to low thousands of loci, screened with a moderate commitment of resources. The advantages are that live resources do not need to be maintained and the labor efforts scale linearly with the increase in the desired number of targets to screen. Parvez et al. used the regular Cas9 nuclease and NHEJ mutagenesis to target coding genes, but there is no reason to think that regulatory elements could not also be targeted in this fashion nor that any of the other CRISPR–Cas modifications could not be used in a MIC-Drop screening strategy, thus opening up new opportunities for questions only answerable in vivo.

Another powerful approach for screening in vivo is a technique coined ‘Perturb-seq’, first developed in cell culture^147^ but eventually adapted to studies in animals. The basic idea is that you combine pooled libraries of sgRNAs with single-cell transcriptional profiling. The sgRNA constructs are detectable as part of the single-cell RNA-sequencing profile, so they are essentially ‘barcoded’ in the assay. In principle, when key regulators of, for example, cell identity are targeted, you should never find targeted cells that have taken on the specific identity. In cell culture, the depth of the screened library can be comprehensive; in animals, it becomes necessary to reduce the screened space significantly. The first in vivo efforts used lentiviral constructs targeting 35 genes believed to be linked to autism risk and the pools were injected into the mouse neocortex in utero^148^. The early postnatal brains were then subjected to single-cell RNA-sequencing and analyzed for emerging patterns of gene expression in different cell types of the neocortex. In this case, the authors found 14 ‘modules’, that is, clusters of genes that varied together, which segregated into different cell types of the neocortex. Researchers further refined the approach by switching to AAV delivery of the guides, allowing for an increased number of targets to be screened and a higher number of total infected cells to be sampled^149,150^.

## CRISPR in gene therapy

The world was shaken when, in 2018, it was announced that twin girls from China had been edited by CRISPR–Cas9 injections in vitro that targeted the HIV receptor CCR5. The work was never published, although the lead researcher had published a (now retracted) ‘Draft ethical principles for therapeutic assisted reproductive technologies’^151^. There was a significant backlash to this act and many potential CRISPR-based therapies were put on pause until clearer ethical standards could be developed. While these manipulations of the human genome at very early embryonic stages are not likely to move forward in the near future, there has been significant progress on therapeutic approaches that can be performed on cells in vitro and then subsequently injected back into the patient. Focusing on diseases that could be alleviated by manipulating cells outside the body eliminates the key hurdle in CRISPR-based therapies: the inability to deliver the riboprotein efficiently to all cells needing treatment.

The first trial of a CRISPR–Cas therapy in humans took place in China^152^. Preclinical data showed that knocking out the PD1 gene in T cells and reinfusion showed enhanced antitumor activity^153^. They took T cells from 17 patients and electroporated the cells with CRISPR–Cas9 complexes targeting PD1. After an average of 25 days, the cells were reinfused into the patients and tracked for disease progression and persistence of edited cells. There were no clinical improvements in any patients, but the edited cells were detectable to 76 weeks postinfusion.

Sickle cell anemia is a disease whose genetic cause has been known for more than 40 years. It is a mutation in the HBB gene that causes protein aggregation, but the first gene therapy was only approved by the Food and Drug Authority (FDA) in 2023. Researchers have shown that an effective approach to diluting the mutant form of HBB is to target a gene, *BCL11A*, whose normal function is to inhibit the expression of fetal forms of hemoglobin^154^. By removing *BCL11A* function, fetal hemoglobin levels rise and alleviate the worst of the mutant HBB symptoms. The treatment was recently approved by the FDA for treatment of sickle cell disease and β-thalassemia^155^. Other blood diseases are likely to have treatments developed soon as they are essentially the only ones where CRISPR–Cas9 edit the cells in vitro. Other diseases will rely on improved delivery systems where editing can be both efficient and widespread in vivo. There are many delivery systems, such as AAV^156^, lentiviral and retroviral vectors^157^, liposome-based nanoparticles^158^ and exosomes^159^. All of these approaches show some promise (see Table 3 for a survey of approaches tested in vivo), but all share the same issues and challenges involved when most, if not all, the cellular genomes in a human body need to be corrected. There will need to be significant improvements in delivery systems to make complete gene correction a reality if it is not done as an early embryo.

**Table 3:** Summary of in vivo base editing applications in genetic disorders.

Disease	Target gene	Mutation	Base editor	Delivery	Notes	Reference
Phenylketonuria	*Pah*	c.1222 C > T p.Arg408Trp	ABE8e-SpRY	Dual AAV8	19–35% A-to-G correction at *Pah* R408W; blood Phe reduced below therapeutic 360 µM, with normal weight and coat color. Brain neurotransmitters normalized when treated neonatally	^189^
Progeria	*Lmna*	c.1824C>T; p.G608G	ABEmax-VRQR	AAV9 systemic delivery	Correction of splicing defect that causes Hutchinson–Gilford progeria syndrome	^82^
Hereditary Hearing Loss	*Tmc1*	c. 545 A > G p.Y182C	AID-CBEmax	AAV delivery to inner-ear injection	~20–30% of cochlear hair cells genotypically corrected; restored inner hair cell mechanotransduction and morphology, with transient improvement of low-frequency hearing at 4 weeks post-treatment	^190^
Usher syndrome type 1 F	*Pcdh15*	c.733 C > T, p.R245X	ABE8e	Dual AAV in cochlea	Restored hearing in a mouse model	^191^
Hereditary hearing loss	*Otof*	c.2485 C > T, p.Q829X	ABE7.10max	AAV injected into the inner ear	Rescued auditory function of mice to near wild-type levels	^192^
Hereditary hearing loss	*Mpzl2*	c.220 C > T, p.Q74X	ABE8eWQ-SpRY	Dual AAVs into the inner ear	Restored hearing function	^193^
Marfan syndrome	*FBN1*	c.7498 T > C, p.C2500R	BE3	Microinjection BE3 mRNA and sgRNA	~89% correction efficiency in heterozygous human embryos using BE3-mediated base editing. Potential for early stage treatment of Marfan syndrome	^194^
Inherited retinal disease	*Rpe65*	c.130 C > T, p.R44X	ABEmax	Subretinal injection of a lentivirus	Corrected a de novo nonsense mutation to restore vision	^195^ /
Retinitis pigmentosa	*Pde6b*	c.1678 C > T, p.R560C	SpRY-ABE8e	Subretinal delivery of dual AAVs	Corrected the pathogenic mutation in the rd10 mouse model of retinitis pigmentosa with up to 49% efficiency, preserving photoreceptor structure and function	^196^
Niemann–Pick disease	*Npc1*	c.3182 T > C p.I1061T	CBE3.9max	Dual AAV9	Improved neuronal survival and extended lifespan of treated mice	^197^
Duchenne muscular dystrophy	*Dmd*	Exon 50 deletion causing frameshift	ABE8e	AAV9	Restored >75% dystrophin in heart; improved muscle function to near wild-type levels.	^198^
Duchenne muscular dystrophy	*Dmd*	Premature stop codon in *mdx*^4*cv*^ mouse model.	iABE-NGA	AAV9	Achieved near-complete dystrophin rescue in heart (~100%) and up to 15% in skeletal muscle fibers at 10 months post-treatment; improved muscle function; low off-target activity and no obvious toxicity	^199^
Duchenne muscular dystrophy	*Dmd*	A > G, premature stop codon corrected to glutamine (TTA > CAA)	ABE7.10	Dual transsplicing adeno-associated viral vector (tsAAV) via intramuscular administration	Restored muscle function in *Dmd* mouse model	^200^

## Future considerations

The number of tools developed using the CRISPR–Cas programmable DNA targeting at the core has exploded in the past decade and no doubt will continue well into the future. We have seen the field move from generating ‘simple’ DSBs to pinpoint conversions of single base pairs with high efficiency. We can also create epigenetic modifications, control RNA transcription and insert large genetic modifications. As the binding targets are so easily modified simply by changing the RNA guide, large-scale screening in different modalities is also possible. These techniques are quite effective but are more challenging to apply to large-scale screening in vivo. This is, however, essential for accelerating our understanding of biological processes that are impossible to study in cell culture. The primary challenge for in vivo studies is the same one that plagues using CRISPR–Cas for gene therapy in humans: the efficient delivery of the enzyme complex to every cell in a living organism is extremely challenging, as is analyzing what happens in the organism after the editing process is complete. In experimental animal (and plant) models where delivery can take place at the one cell stage, the next most important issue is speed and efficiency of the edits. The earlier the modifications occur, the less mosaic the animal will be and the higher the probability the analysis can take place in the first generation instead of having to go through a ‘purifying’ breeding regimen to outcross and/or incross the modification, delaying the results and increasing the costs of screening. We have seen huge improvements on the efficiency side, with many of the techniques obtaining biallelic conversion in both mouse and zebrafish-injected embryos. We are on the cusp of a new and exciting time for the analysis of disease alleles in vertebrate model systems and broad functional genomics in vivo.
